# Decreasing the Effective Thermal Conductivity in Glass Supported Thermoelectric Layers

**DOI:** 10.1371/journal.pone.0151708

**Published:** 2016-03-16

**Authors:** Kevin Bethke, Virgil Andrei, Klaus Rademann

**Affiliations:** Department of Chemistry, Humboldt-Universität zu Berlin, Berlin, Germany; Tsinghua University, CHINA

## Abstract

As thermoelectric devices begin to make their way into commercial applications, the emphasis is put on decreasing the thermal conductivity. In this purely theoretical study, finite element analysis is used to determine the effect of a supporting material on the thermal conductivity of a thermoelectric module. The simulations illustrate the heat transfer along a sample, consisting from Cu, Cu_2_O and PbTe thermoelectric layers on a 1 mm thick Pyrex glass substrate. The influence of two different types of heating, at a constant temperature and at a constant heat flux, is also investigated. It is revealed that the presence of a supporting material plays an important role on lowering the effective thermal conductivity of the layer-substrate ensemble. By using thinner thermoelectric layers the effective thermal conductivity is further reduced, almost down to the value of the glass substrate. As a result, the temperature gradient becomes steeper for a fixed heating temperature, which allows the production of devices with improved performance under certain conditions. Based on the simulation results, we also propose a model for a robust thin film thermoelectric device. With this suggestion, we invite the thermoelectric community to prove the applicability of the presented concept for practical purposes.

## Introduction

Thermoelectric devices are widely used in a broad range of fields, where stability and reproducibility of the response over a long lifespan is needed. Examples of applications range from thermonuclear batteries for remote locations and deep space exploration, [1, 2] to heating and cooling elements for polymerase chain reaction (PCR) devices.[3, 4] The wide acceptance of thermoelectric devices for energy recuperation is largely dependent on the increase of performance and lowering of the production costs. Nevertheless, notable steps are made in these directions, [5–7] which bring industrial scale applications closer to reality.[8–10] One prominent example is the prototype developed by the BMW group for mass introduction in hot car exhausts, which generates directly electricity from the waste heat.[11]

The challenging task for all research in the field of thermoelectrics is the improvement of the figure of merit (ZT), which is related to the efficiency of the energy conversion. This implies the optimization of the constituting parameters: the Seebeck coefficient (S), the electrical conductivity (*σ*) and the thermal conductivity (*κ*). The relationship between ZT and these material properties is presented in Eq (1), where T is the temperature, and PF represents the power factor.

$$\begin{array}{c} ZT=\frac{\sigma S^{2}}{\kappa }T=\frac{PF}{\kappa }T \end{array}$$ (1)

Looking at Eq (1), it becomes clear that lowering the thermal conductivity increases the figure of merit, which consequently leads to a higher efficiency. The improved performance for lower *κ* can be also explained in another simple way. A higher *κ* translates to a greater heat transfer through a thermoelectric generator, which results in a practically reduced temperature difference between the two ends. Since the magnitude of the produced voltage is directly dependent on the scale of the temperature difference, the performance also declines. This interpretation becomes especially important in case of thinner thermoelectric devices, where the initial heat gradient is often lost over time. [12–15]

Nevertheless, the thermoelectric performance of one material depends on all three named parameters: S, *σ* and *κ*. These properties vary over a wide range, making different types of materials available for various applications. On the one side there are metals, which possess high thermal and electrical conductivities and small Seebeck coefficients, such as copper and silver. Different types of alloys are often used in thermoelectric applications, such as type K thermocouple sensors for electronic thermometers, due to the reproducibility of the results. These alloys present a higher thermoelectric power in comparison with pure metals, leading to a better sensitivity.[16, 17] On the other side, there are semiconductors, such as transition metal oxides, which present high Seebeck coefficients but low electrical conductivities.[18, 19] These compounds are currently investigated as an environmentally friendly alternative to existing thermoelectric materials.[20–26] Generally speaking, chalcogenides and especially tellurides are currently the most widespread thermoelectric materials. Such compounds manage to achieve a compromise between a high Seebeck coefficient and a high electrical conductivity, maximizing the figure of merit.[27–31] Bismuth telluride (Bi_2_Te_3_) and lead telluride (PbTe) are good examples for thermoelectric materials found in commercial applications.[32–34] Nevertheless, these elements are toxic and rare, therefore alternatives, such as oxytellurides, [35, 36] oxides [37, 38], organic polymers [39–42] or composites [23, 43, 44] are still needed.

It is generally accepted that the use of nanometer scale thermoelectric devices drastically reduces the costs of production, due to the small amount of raw material needed. Furthermore, there is also a theoretical background that low dimensional materials present enhanced thermoelectric properties.[24, 45–47] This low dimensionality can be for example achieved by depositing nanometer thick films of desired materials on various substrates, such as glass or flexible polymer layers.[48–50] However, the influence of the substrate on these properties is often neglected in thin film thermoelectric measurements. Since the mass of the substrate is significantly higher than the mass of the thermoelectric material, its thermal conductivity should play an important role in the heat transfer through the thin film, altering the efficiency in real life applications. This effect is what we define as the effective thermal conductivity of a supported thin film, as opposed to the actual thermal conductivity of its bulk material.

In order to quantify the influence of the substrate on the thermal conductivity, extensive experiments in the nanometer region are normally needed. Therefore, theoretical and computational studies are often performed on complex systems.[51–57] This motivated us to study heat transfer phenomena from a fundamental point of view. By depicting the heat flow within a basic experimental setup, we could deduct general conclusions, which could be applied in various other cases.

In this work we present the simulation results of our heat conductivity studies on free standing and supported copper, copper(I) oxide (Cu_2_O) and lead telluride thin films, as representatives for a wide range of materials, spanning from conductors to semiconductors. To better illustrate the changes in temperature within various thin samples, we employ the versatile finite element method, [58, 59] which has proven itself in most various research fields.[60–63] In addition to providing qualitative information on the effective thermal conductivity, this method also helps illustrating the time dependence of the temperature, which is often the case in practical applications, such as energy recovery systems for car exhaust heat.[57] Using the comprehensive information obtained, we further design a model for thin film thermoelectric devices.

## Materials and Methods

All simulations are done using the software Comsol Multiphysics 4.4, whereas the curves are plotted in Origin. In the study, thin films of thermoelectric materials (Cu, Cu_2_O and PbTe) are modeled on a glass microscope slide as a supporting material. Three important parameters for the simulations, the thermal conductivity (*κ*), the heat capacity at constant pressure (*C*_*P*_) and the density (*ρ*) of the mentioned substances are presented in Table 1, along with their thermoelectric properties.[16, 18] The values are mostly given for the temperature of 293 K.

**Table 1:** Several material parameters of copper, copper(I) oxide and lead telluride at 293 K.

Sample	S [*μ*V K^−1^]	*σ* [S m^−1^]	*κ* [W m^−1^K^−1^]	PF [*μ*W m^−1^K^−2^]	ZT [-]	*C*_*P*_ [J kg^−1^K^−1^]	*ρ* [kg m^−3^]
Cu	1.83 ^*a*^	5.99⋅10^7^ ^*b*^	397 ^*b*^	201	1.48⋅10^−4^	383 ^*b*^	8940 ^*b*^
Cu_2_O	1100 ^*c*^	2.24⋅10^−3^ ^*c*^	6.28 ^*c*^	2.71⋅10^−3^	1.26⋅10^−7^	436 ^*c*^	6000 ^*b*^
PbTe	187 ^*d*^	6.10⋅10^4^ ^*d*^	1.46 ^*d*^	2132	0.43	151 ^*d*^	8160 ^*d*^

^a^ - Value at 300 K, taken from Cusack and Kendall.[16]

^b^ - Data obtained using the fitted functions in the Comsol Multiphysics database (see S1 Table).

^c^ - Data taken from the works of Vogt.[18] *σ* is given at 298K and *κ* at 273K.

^d^ - Given values in the Comsol Multiphysics database.

The parameters are needed to describe the material’s thermoelectric properties and to simulate the heat transfer. The power factors (PF) and the figures of merit (ZT) are calculated at 293K from the given S, *σ* and *κ* values.

Pyrex is the chosen type of glass, due to its broad availability and use in laboratory investigations. S1 Fig presents the thermal conductivity and thermal capacity of the Pyrex glass within the temperature range of 270K to 400K. The fitted functions of the curves are also presented in S1 Table, as taken from Comsol Multiphysics. Both parameters undergo significant change in this interval, increasing from 1.05 Wm^−1^K^−1^ to 1.25 Wm^−1^K^−1^ in case of the thermal conductivity, and from 700 Jkg^−1^K^−1^ to 900 Jkg^−1^K^−1^ in case of the thermal capacity. The density of the glass varies minimal in this range, between 2227–2231 kgm^−3^.

**Fig 1:**
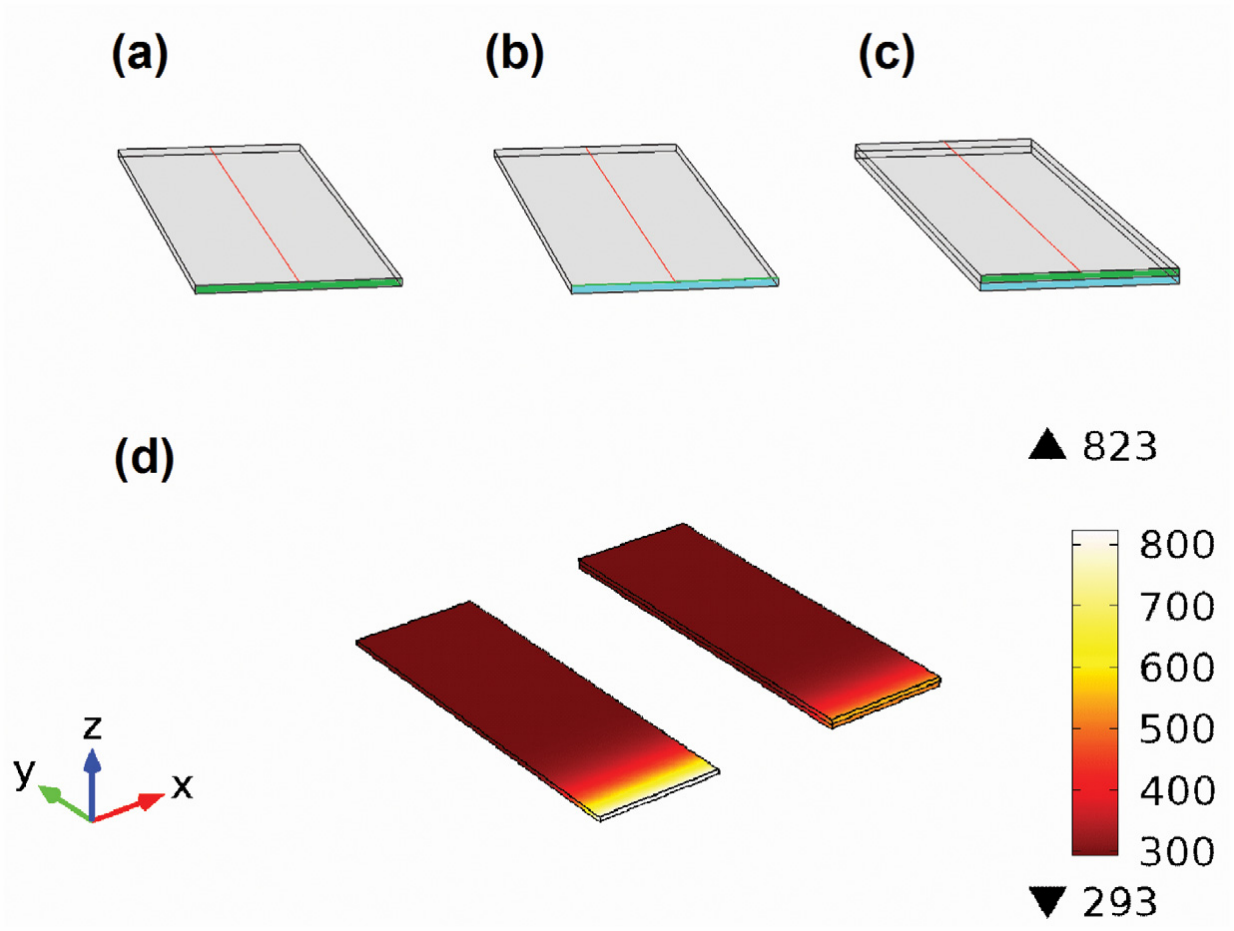
Various views of the modeled thin layers. (a)-(c) Wireframe model with red cut lines at which the temperature is measured, shown for: (a) free standing films, (b) thin films supported on Pyrex glass and (c) thick films supported on Pyrex glass. The heating contact interface is represented either by the green area (for the thermoelectric layer) or the blue area (for the Pyrex glass); the grey surfaces are thermally isolated from the outside. (d) Visualization of the temperature distribution for a 1 mm thick PbTe layer (left) and for the same layer on a Pyrex substrate (right). A heat flux of 10^5^ Wm^−2^ is applied to the front face of each PbTe film. The temperature distribution is illustrated after 40seconds of heating.

For the glass substrate the dimensions of a standard microscope slide are used, 25.4 mm × 76.2 mm × 1.0 mm. Thin and thick layers are modeled as rectangular solids with the same width and depth as the supporting material. For graphs the temperature is measured along the y axis, on the top surface of the thermoelectric thin layer, in the middle of it. This corresponds to the red lines in Fig 1(a)–1(c) and S6 Fig.

Eq (2) is used in Comsol Multiphysics for the calculations of heat transfer in solids, where *ρ* is the density, *C*_*P*_ is the heat capacity at constant pressure, T is the absolute temperature, u the velocity field, *κ* the thermal conductivity and Q the heat source. The simulations contain various approximations, in order to reduce the computing time. The chosen materials are solids, which eliminates effects, such as convection. The cooling through the surrounding air is further neglected (all outer surfaces except one are thermally isolated), thereby removing a multitude of other parameters (convection, flow, composition and pressure, to name a few), which would have complicated the results. This is also the case in practical applications since most devices are optimized to minimize convectional heat loss by air flows. Consequently, the given approximations enhance the speed and reproducibility of the simulations, without significantly altering the thermal transfer. The obtained basic model allows us to draw general conclusions on the heat transfer within thin layers.

$$\begin{array}{c} \rho C_{P}\frac{\partial T}{\partial t}+\rho C_{P}u\cdot \nabla T=\nabla \cdot \left(\kappa \nabla T\right)+Q \end{array}$$ (2)

Another important approximation is the absence of the size effects and interfacial thermal resistance from our classical heat diffusion model, which can also influence the thermal conductivity of nanometer thin films. As explained in detail at a later point, the nanoscale size effects only strengthen our conclusions, by lowering the thermal conductivity of the thin films even more.[64, 65] Moreover, the interfacial thermal resistance is strongly dependent on experimental conditions and cannot be precisely evaluated by well-known physical models (acoustic mismatch and diffuse mismatch); therefore its influence can only be qualitatively discussed. [66]

**Fig 2:**
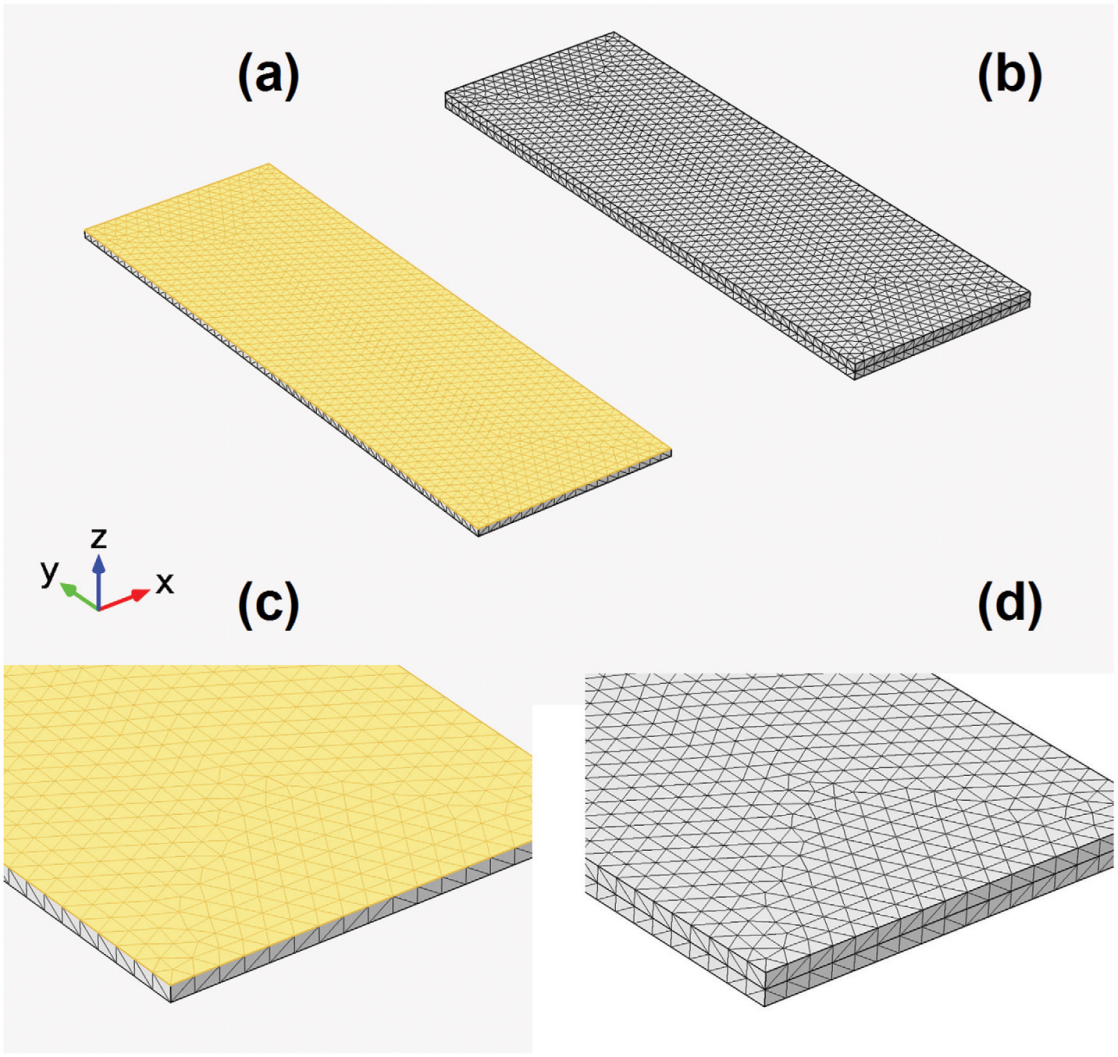
Meshes created for the finite element method simulations of the heat transfer in solids. The meshes of the supported thermoelectric layers are shown for: (a) the 100 mm thin film, (b) the 1 mm thick layer. Detailed views of both cases are also given in frames (c) and (d). The swept mesh of the nanometer thin layer is highlighted in yellow.

For the computation of time dependent heat transfer studies, the finite element method is employed. For mesh elements tetrahedrons are chosen, except for thin features where a swept mesh is needed. Accordingly, the thickness of the swept mesh elements is restricted to that of the thin film. In case of the tetrahedrons, the maximum element size is set to 4.19⋅10^−3^m and the minimum element size to 3.05⋅10^−4^m. The maximum element growth rate is set to 1.4, the curvature factor to 0.4 and the resolution at narrow regions to 0.7. The relative repair distance has a value of 1⋅10^−8^m. Images of the various meshes are presented in Fig 2, where the swept mesh is highlighted in yellow. As shown there, the 100nm thin elements mirror the triangular pattern present at the supporting layer’s surface.

The time dependent studies are made from 0 to 40 seconds in steps of 0.1s. The front side of the simulated solid (either green or blue areas in Fig 1(a)–1(c)) is the only surface heated during this period, whereas the other faces are thermally isolated. Consequently, the simulated system resembles the general case of thermal resistances in parallel. The initial temperature of the body is set to 293K. The heating occurs in two ways, either by maintaining a constant temperature of 393 K at one end, or by applying a constant heat flux of q_0_ = 10^5^ Wm^−2^. The first case is better suited for practical applications, corresponding to the heating through a thermostat (such as an industrial waste heat exchanger), while the latter is more of theoretical interest, giving a measure of the thermodynamic device efficiency.

A selected result of our study is depicted in Fig 1(d), which visualizes the temperature distribution in a free standing and a Pyrex glass supported PbTe layer after 40 seconds of constant heating at the front face with 10^5^ Wm^−2^. Another example can be also found online in S2 Fig, showing the time dependence of the temperature profile for a supported PbTe layer.

## Results and Discussion

**Fig 3:**
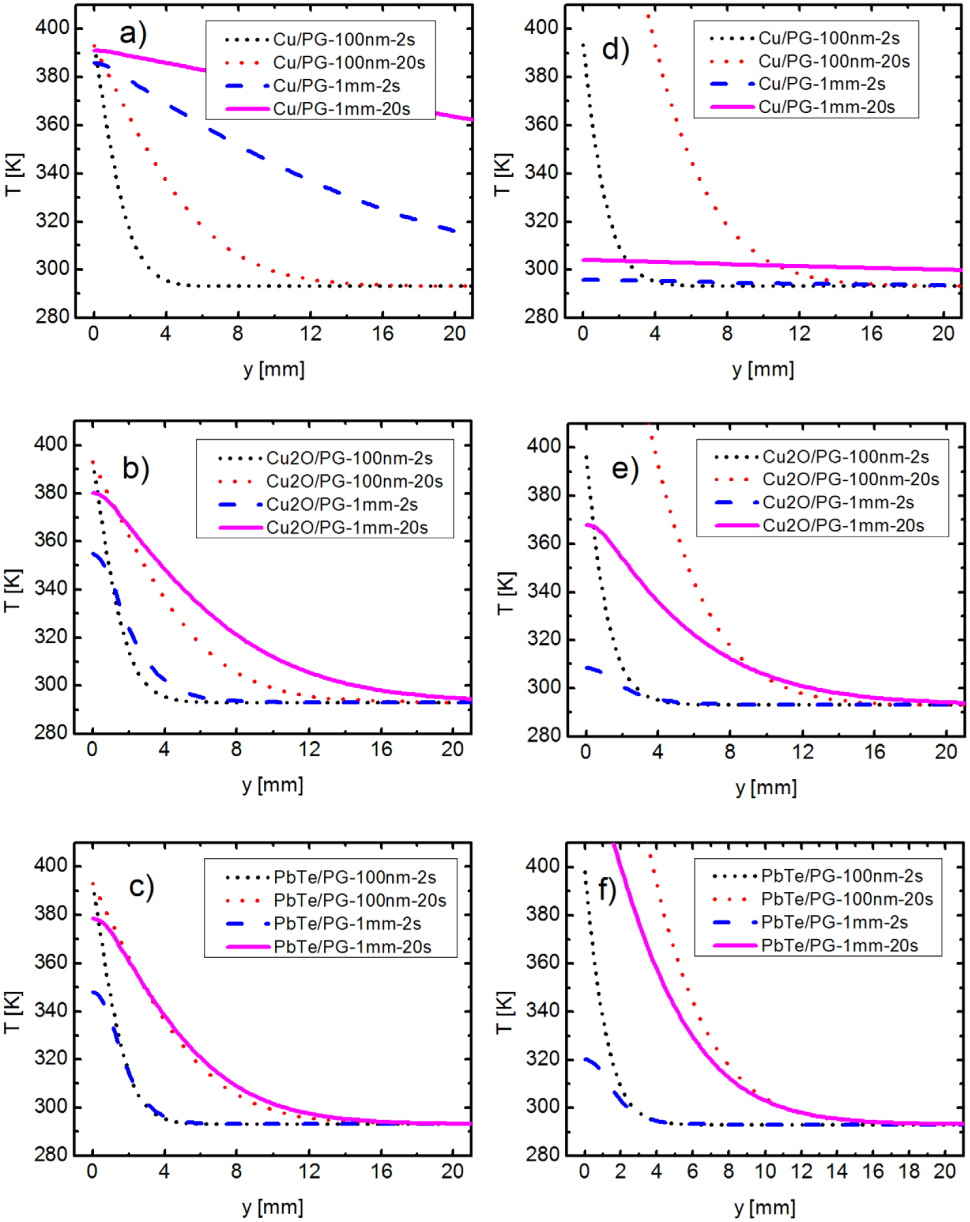
Comparison of a 100 nm thin film with a 1 mm thick layer, both supported on a glass slide. The temperature is kept constant at the front face of the Pyrex glass (PG) for: (a) Cu, (b) Cu_2_O, (c) PbTe. The heat flux is maintained constant at the front face of the Pyrex glass for: (d) Cu, (e) Cu_2_O, (f) PbTe.

**Fig 4:**
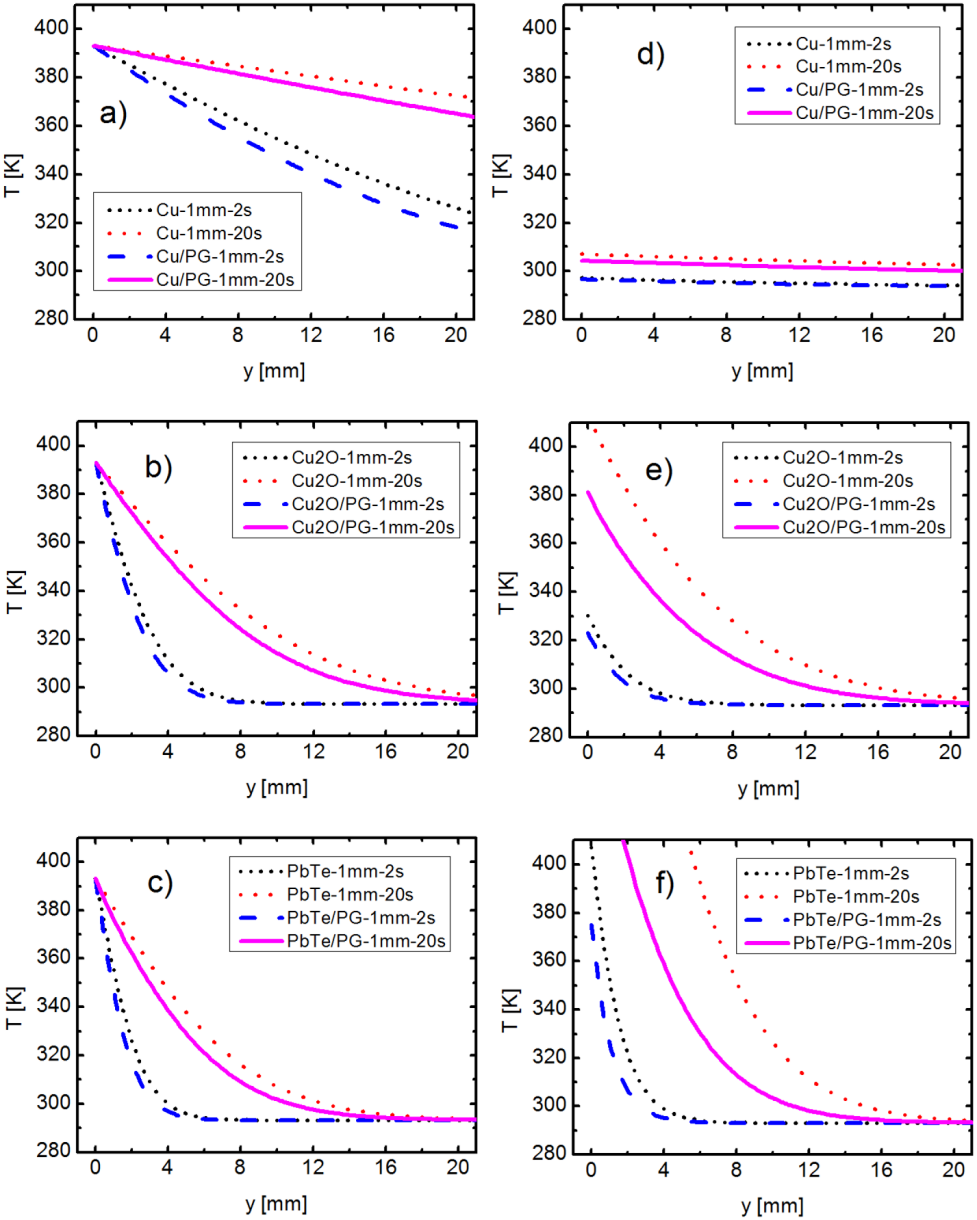
Comparison of a free standing 1 mm thick layer and a 1 mm layer on a microscope slide. The temperature is kept constant at the front face of the Pyrex glass (PG) for: (a) Cu, (b) Cu_2_O, (c) PbTe. The heat flux is maintained constant at the front face of the Pyrex glass for: (d) Cu, (e) Cu_2_O, (f) PbTe.

As mentioned above, the thermal conductivity and the heat capacity of the chosen materials have a major role on the overall heat transfer within a sample. In this classical model, two other parameters influence the heat transfer as well: the thickness of the thin layer and the presence or absence of a substrate. The variation of the different parameters investigated is illustrated below, in Table 2, as well as in Figs 3 and 4. Here, the differences in the temperature gradient along various thin layers are investigated, as a measure of the effective thermal conductivity. The y axis is plotted from 0 mm to 20 mm and the temperature axis from 280 K to 410 K, in order to permit comparisons between the different curves and to improve the visibility. Full range plots, with depth between 0-76.2 mm and complete temperature scales, are presented online in S3 and S4 Figs.

**Table 2:** Overview of the conducted experiments and their corresponding figures.

Figure	Heated surface	Heat source	Thermoelectric material	Thickness	Substrate
2(a)	Pyrex	*T*_0_ = 373K	Cu	100nm	Pyrex
2(a)	Pyrex	*T*_0_ = 373K	Cu	1mm	Pyrex
2(b)	Pyrex	*T*_0_ = 373K	Cu_2_O	100nm	Pyrex
2(b)	Pyrex	*T*_0_ = 373K	Cu_2_O	1mm	Pyrex
2(c)	Pyrex	*T*_0_ = 373K	PbTe	100nm	Pyrex
2(c)	Pyrex	*T*_0_ = 373K	PbTe	1mm	Pyrex
2(d)	Pyrex	*q*_0_ = 10^5^Wm^−2^	Cu	100nm	Pyrex
2(d)	Pyrex	*q*_0_ = 10^5^Wm^−2^	Cu	1mm	Pyrex
2(e)	Pyrex	*q*_0_ = 10^5^Wm^−2^	Cu_2_O	100nm	Pyrex
2(e)	Pyrex	*q*_0_ = 10^5^Wm^−2^	Cu_2_O	1mm	Pyrex
2(f)	Pyrex	*q*_0_ = 10^5^Wm^−2^	PbTe	100nm	Pyrex
2(f)	Pyrex	*q*_0_ = 10^5^Wm^−2^	PbTe	1mm	Pyrex
3(a)	thin layer	*T*_0_ = 373K	Cu	1mm	-
3(a)	thin layer	*T*_0_ = 373K	Cu	1mm	Pyrex
3(b)	thin layer	*T*_0_ = 373K	Cu_2_O	1mm	-
3(b)	thin layer	*T*_0_ = 373K	Cu_2_O	1mm	Pyrex
3(c)	thin layer	*T*_0_ = 373K	PbTe	1mm	-
3(c)	thin layer	*T*_0_ = 373K	PbTe	1mm	Pyrex
3(d)	thin layer	*q*_0_ = 10^5^Wm^−2^	Cu	1mm	-
3(d)	thin layer	*q*_0_ = 10^5^Wm^−2^	Cu	1mm	Pyrex
3(e)	thin layer	*q*_0_ = 10^5^Wm^−2^	Cu_2_O	1mm	-
3(e)	thin layer	*q*_0_ = 10^5^Wm^−2^	Cu_2_O	1mm	Pyrex
3(f)	thin layer	*q*_0_ = 10^5^Wm^−2^	PbTe	1mm	-
3(f)	thin layer	*q*_0_ = 10^5^Wm^−2^	PbTe	1mm	Pyrex

Although the computed time dependent studies span over an interval of 40seconds, it is in the interest of the reader to present only eloquent results, which are not redundant. Therefore, the temperature profile along the surface of the thin layers is only presented at two arbitrarily chosen times, after 2s and respectively after 20s. At these times, the simulated curves differ significantly from each other, so that a change over time can be clearly observed. Nevertheless, the choice of the temperature profiles does not alter the validity of the deduced relationships, i.e. another pair of curves would reveal the same conclusions. This can be also verified by looking at Fig 1(d) and S2E Fig, which illustrate various heat distributions after 40s. To further emphasize that, the computed profiles of some supported PbTe layers are also given online in S7 Fig, between 1-40s, in 1s steps. Besides the gradual increase in temperature, no other difference is observed.

### Comparison of a 100 nm thin film with a 1 mm thick layer, both supported on a microscope slide

For the first investigations, only the front side of the Pyrex glass is heated (coloured blue in Fig 1(b) and 1(c)). The front side of the thermoelectric layer (green) remains thermally isolated, like the rest outer surfaces. As a result, a smaller temperature is achieved at the upper edge of the thermoelectric film (y = 0 mm in Fig 3(a) to Fig 3(c)). The distribution of temperature along two directions, namely the height and the length of the ensemble, is also clearly visible in S2A and S2B Fig. Due to this 2D heat distribution, the system cannot be precisely described using an analytical 1D model. This could also be the more realistic representation since the thin layer will not always be in perfect contact with the heating source (depending on the manufacturing process and material properties). For example, the heating is mostly made through the substrate in thin film thermoelectric measurements, where the thermal contact of the thin film with the heater is not ideal.

Fig 3(a), 3(b) and 3(c) illustrate the case, when the temperature at the heating end is maintained constant, at 393K. The influence of the thermal conductivity on the heat transfer is most noticeable in case of the 1 mm thick copper layer (Fig 3(a)), in comparison with the corresponding plots of copper(I) oxide (Fig 3(b)), and lead telluride (Fig 3(c)). While the last two achieve already a temperature of approximately 293K at y = 20 mm after 20s, the copper layer presents a value of 363K at the same point. Even at the opposing face, the copper thick film still reaches 324K, due to its high thermal conductivity (see S3 Fig). This behavior is visible even after 2s.

The temperature profiles of the 100nm layers are very similar for the three materials, due to their small mass in comparison with that of the supporting material. At the same time, they resemble the profile of the pure substrate, which is given in S5 Fig. Therefore, the thermal properties are largely dictated by the supporting material. This observation is also in accordance with the 1D model of thermal resistances in parallel, where the effective thermal conductivity approaches the value of the substrate (see S1 Text and the corresponding S6 Fig for the mathematical demonstration). The small thermal conductivity of the substrate has then a further consequence, namely the temperature gradient is steeper. This translates in a shorter distance required to reach 293K than in the case of thicker thermoelectric layers.

Fig 3(d) to Fig 3(f) illustrate a different approach to this problem. This time, a constant heat flux of 10^5^Wm^−2^ is sent through the front face of the glass substrate, which causes different temperature responses. The dimensions of the heated surface are 25.4 mm × 1.0 mm, therefore the surface has an area of 25.4⋅10^−6^m^2^, corresponding to a heating power of 2.54W.

As in case of the constant temperature at one end, the influence of the thin thermoelectric layer on the overall heat transfer is negligible. The small thermal conductivity of the glass substrate means that the heat remains localized (293K are reached within 20 mm of the heating source). This leads to a significant increase in temperature at one end, up to 595K for PbTe (see S3 Fig).

In case of the 1 mm thick films, their thermal properties play again an important role. The temperature gradient obtained in this case is lower in comparison with the 100nm films. This can be explained by the fact that the mass of the layer-substrate ensemble is increased, while the heating power remains constant at 2.54W. Due to its high thermal conductivity, copper just dissipates the received heat along its body; therefore the temperature gradient is drastically reduced. As a result, a difference of just 9K is obtained after 20s between the two ends of the sample. This makes thick copper layers especially unsuitable for thermoelectric applications, where the temperature gradient should remain high. On the other side, the supported 1 mm PbTe film best conforms to this criterion, producing the highest temperature difference from the available heat flux, 142K, but also cooling to 293K over the smallest distance, approximately 17mm.

### Comparison of a free standing 1 mm thick layer with a 1 mm layer on a microscope slide

As seen above, the thermal properties of thermoelectric thin films are almost entirely determined by the properties of the supporting material. Therefore, thick layers of 1 mm are investigated in the current section, to make the differences between temperature profiles better noticeable. These layers are modeled either alone (see Fig 1(a)), or on a Pyrex glass (Fig 1(c)), to illustrate the influence of the substrate on the effective thermal conductivity. This time, only the front side of the thermoelectric materials (depicted green in Fig 1) is heated, while the outer Pyrex surface (blue) stays thermally isolated.

The changed simulation conditions have an effect on the maximum temperature achieved in Fig 4(a), for Cu, Fig 4(b), for Cu_2_O, and Fig 4(c), for PbTe. It is no wonder that 393K is reached in all three cases since the temperature at y = 0 mm is constrained to this value. On this occasion, the vertical temperature gradient is formed over the height of the substrate layer. The results from the previous section also remain valid here. The copper layer fails again to reach 293K across the length of the glass slide (326K at y = 76.2 mm after 20s, see S4 Fig), while the lead telluride is a slightly better thermal insulator than copper(I) oxide. Nevertheless, a reduction of the effective thermal conductivity is again visible in case of the supported films, from the lower temperatures achieved in comparison with the free standing layers. This qualitative observation is also confirmed by the similar results obtained for the related one dimensional case (see S1 Text).

Fig 4(d), 4(e) and 4(f) further present the temperature distribution within the three thick films, when a constant heat flux of 10^5^Wm^−2^ is used. The apparently unusual profile of the curve for copper can be once more explained through its high thermal conductivity and heat capacity, which lead to a fast distribution of the heat throughout the sample. This is also valid for the thick supported layer, where the heat transport through the metal is far superior to that through the Pyrex glass. As such, the temperature difference along the sample is approximately 9K for both the free standing and the supported copper layer. Consequently, the influence of the glass substrate is minimal. These results from Figs 3(d) and 4(d) indicate the limits of the simple 1D model, which cannot accurately describe the heat transfer for thicker supported metallic layers, due to their far higher thermal conductivity in comparison to that of the glass substrate. Otherwise, the lead telluride presents itself again as the material of choice for thermoelectric applications, building the highest temperature difference, 369K, over the shortest distance, approximately 22mm.

The difference between the free standing and supported layers is quite small in Fig 4(b)–4(d), due to the larger thickness (i.e. also higher mass) of the thermoelectric layer, whose front surface is directly heated. Since the active materials have higher thermal conductivities than the substrate, the heat tends to diffuse more directly to the colder side through them. At the same time, the lateral heat exchange between the thermoelectric and the supporting layer becomes lower, which means the temperature distribution changes less considerably. This is best observed in Fig 4(d), where almost all heat is conducted through the copper layer; therefore the temperature difference becomes minimal. For thinner layers, the direct heat transfer through the active material becomes more difficult, while the heat exchange with the supporting material gains more importance. A more physical explanation is given by the propagation of the phonons in a medium, which is more challenging for thinner materials, when the layer’s thickness is below their mean free path. The boundary scattering, which depends on the supporting layer, also increases considerably for such thin films. Therefore, the difference between free standing and supported layers increases, when the thickness of the thermoelectric film decreases. This is also seen when comparing Figs 3 and 4, and further confirmed by the theoretically expected lower effective thermal conductivity (see S1 Text for the 1D model of resistances in parallel).

Another notable observation about the heat transfer can be made by looking at the plots for free standing thermoelectric layers, in Fig 4(d)–4(f). The maximum temperature gradients are not only increased with lower *κ*, but they are also higher than the ones for supported layers. This decrease in temperature for the two-component system can be explained in a couple of ways. On one side, the same argument can be used as in case of Fig 3(d)–3(f), namely a constant heating power is used for a higher mass. From a thermodynamic point of view, the additional heat flow over the supporting material generates entropy and no work, thus increasing the irreversibility of the device even more and reducing the theoretical achievable efficiency. As a result, this approach is less suitable for thermoelectric applications in case of a constant heat flux, where a maximization of the temperature difference is desired.

### Factors influencing the thermoelectric efficiency of supported thin films

As mentioned in the introduction, our FEM simulations are conducted using the classical heat diffusion model and the bulk properties of the solids. This allows us to draw general conclusions on how the layer’s thickness, composition and substrate affect the heat transfer, without overcomplicating our systems. Nevertheless, the thermal resistance of a nanometer thin layer can differ from that of its bulk material. For real life applications, two other important factors must be further taken into consideration. On one side, the size effects have a further influence on the effective thermal conductivity of the thin layer, leading to a ballistic-diffusive heat transfer.[64, 65] On the other side, the interfacial thermal resistance (thermal contact resistance or thermal boundary resistance) also affects the heat transmission between the film and the glass substrate.[66] In this subsection, we qualitatively describe the influence of these factors on the efficiency of our supported thin films. These effects are also discussed in detail in several other related papers, which follow a more theoretical approach.[67–77]

The size effects at the nanoscale can be divided in two main parts: the ballistic heat transport (where the heat carrier mean free path is higher or comparable to the layer’s thickness) and the lateral phonon-boundary scattering (which diminishes the heat flux).[64, 65] In both cases, their presence leads to a further reduction of the thin layer’s *κ*, which then favors an even lower effective thermal conductivity.[65] Nevertheless, special care must be also given to the ballistic heat transport, which is known to cause temperature jumps at the contact boundaries with the heat sources, that might reduce the actual temperature gradient obtained.[65] Otherwise, the presence of electrons, along the phonons, could also affect the obtained temperature difference in very conductive materials. The influence of those additional heat carriers can be best observed in case of the simulated thicker copper layers.

**Fig 5:**
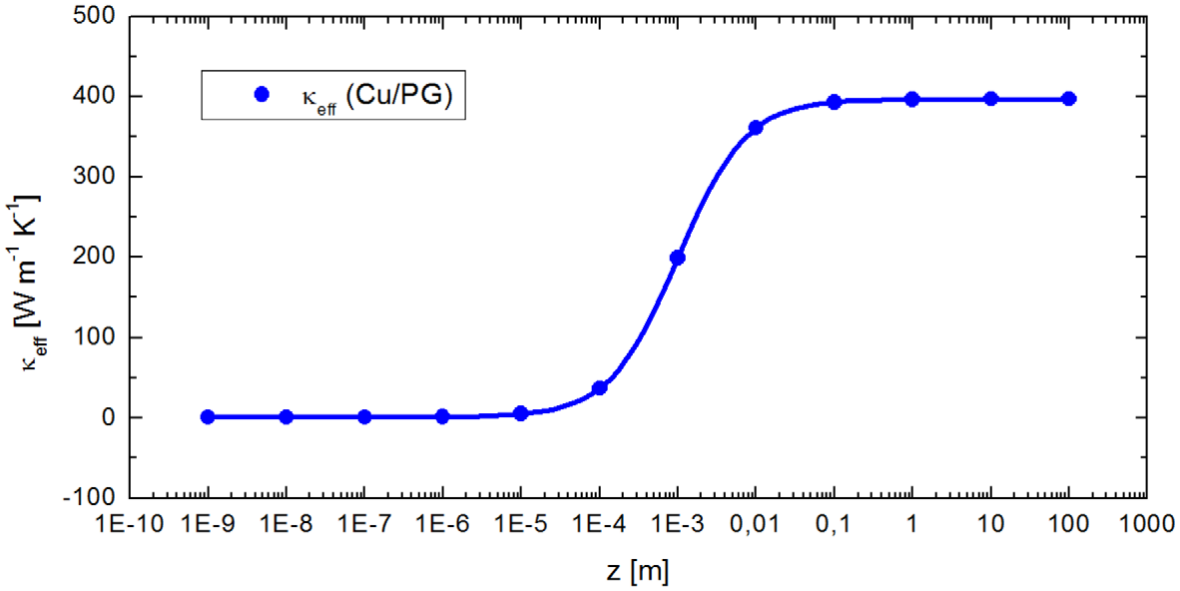
Theoretical effective thermal conductivity of a supported copper layer. The points are calculated using Eq (S3) from S1 Text, which is derived from the 1D model of thermal resistances in parallel. Here, the thickness of the copper layer is varied, while that of the Pyrex glass remains constant (1 mm).

Nevertheless, although such effects may play an additional role for our simulated 100 nm thick thermoelectric layers, this does not make our general conclusions less valid for other thin films. For example, the size effects are expected to have a smaller influence at the micrometer scale since the heat carrier mean free path (MFP) is significantly shorter than the thickness of the layers. This can be also seen from Fig 5, where the dependence of the effective thermal conductivity *κ*_*eff*_ on a copper layer’s thickness is plotted, using the 1D model of electrical resistances in parallel (see Eq (S3) from S1 Text). There, *κ*_*eff*_ approaches the thermal conductivity of glass (around 1.1Wm^−1^K^−1^ at 293K), when the copper layer is only 100 times thinner than the substrate, namely 10 *μ*m thick. In comparison, the phonon MFP is only 40-300nm for bulk silicon at room temperature, [65, 76, 78] and below 100nm for PbTe.[79, 80] Accordingly, the mean free paths of the phonons are also well below 10 *μ*m for many other samples, which means the heat transport is mainly diffusive (corresponding to the classical model).

At the same time, the interfacial thermal resistance can also influence the temperature gradient along the sample length. This is especially relevant in the case, when only the substrate is heated (i.e. the thermoelectric layer is heated indirectly). Although this contact resistance has been mostly investigated for helium-solid interfaces (Kapitza resistance)[67] at very low temperatures (typically 0.1-1K), its two main physical models (acoustic mismatch and diffuse mismatch) have been also applied to solid-solid interfaces.[66, 70] From these two, the diffuse mismatch model should be theoretically more suitable for explaining the thermal boundary resistance at higher temperatures, due to its higher scattering at the interfaces. Nevertheless, both models have failed to explain the interfacial thermal resistance at temperatures above 30K, with theoretical values about one order of magnitude below the experimental ones. These large differences, obtained by various research groups (for different, or even the same substances), have revealed the dependence of the actual values on the sample preparation, purity and surface treatment.[66] As such, although this effect is certainly present, its measure cannot be accurately quantified by either experimental or simulated results since it depends strongly on the experimental conditions. Accordingly, it seems more reasonable from our perspective to neglect it throughout our simulations, in order to enhance the general character of our results. In this case, the contact surfaces of the two layers are considered perfect, with no defects or irregularities. Nevertheless, it should be mentioned that this boundary resistance can be diminished, by obtaining more homogeneous surfaces.

Moreover, the effect of the substrate on the electrical conductivity must be also discussed since *σ* is just as important as *κ* for the improvement of the figure of merit. If we assign the bulk properties to the thermoelectric film, its electrical conductivity would remain constant, regardless of the thin layers’ thickness or width (only the measured resistance would then vary). Since the electrical conductivity of Pyrex glass (6.77⋅10^−18^ Sm^−1^ at 293K, obtained from the Comsol fits) is more than 10 orders of magnitude below that of typical thermoelectric materials (PbTe 6.10⋅10^4^ Sm^−1^), we can confidently say that the electrical transport occurs only through the conductive film. As such, the insulating glass does not contribute to an effective electrical conductivity, in contrast to the thermal transport, where its phonons ensure some heat transfer. This remains valid for every layer’s thickness (i.e. even for the 100 nm films), as long as the substrate is a good electrical insulator. Otherwise, the effect of the size reduction on the electrical transport is weaker than in case of the phonons since the confined electrons can still move freely within the x-y plane. More precisely, the charge carrier mobility is maintained constant along that plane, since there is no interface scattering between the thermoelectric film and the insulating layer.[81] This is also the reason why higher ZTs of up to 7 can be theoretically expected for nanolayered compounds.[81]

As shown above, the thermal and electrical conductivity of supported thin layers can favor an even higher thermoelectric efficiency than that expected from our results, when taking nanoscale effects into account. Nevertheless, the improvements revealed by our simulations remain valid for even thicker supported layers, which can reach up to around 10 *μ*m, according to the 1D model.

### Design of a thermoelectric device using supported thin films

Using the results obtained for the planar layers above, it is possible to predict the thermoelectric properties of more complex systems. In order to design, for example, a thin layer based thermoelectric generator (TEG) with a higher performance, several factors need to be taken into account. The use of thinner layers on a substrate with a low *κ* is more favorable since the temperature difference along a thermoelectric leg is increased. This is most noticeable for metals with high *κ*, such as copper. Simultaneously, the costs for the TEGs are reduced since less thermoelectric material is required. The gradient becomes also steeper in case of heating through a thermostat, when the temperature at an end remains constant; therefore the TEGs can become thinner. This lowers further the costs of production, by reducing the amount of supporting material used.

At this point, we have to discuss the difference between thermodynamic efficiency and device performance in practical applications. The fact, that these two distinct concepts are often mistaken as one, has already led to elaborate discussions in the literature.[53] On one side, the thermodynamic efficiency of a thermoelectric thin layer is reduced in the presence of a substrate, as illustrated by the reduction of the temperature difference at a constant heat flux (see Fig 4(d)–4(f)). On the other side, the most encountered heating sources (from the common household radiators to industrial heat exchangers) maintain a constant temperature, therefore acting as thermostats. This time, the emphasis is put on the length needed for cooling, instead of the supplementary energy required to preserve the temperature. Consequently, the use of supported thin films is actually favorable in these cases, as presented above, leading to an improved device performance.

**Fig 6:**
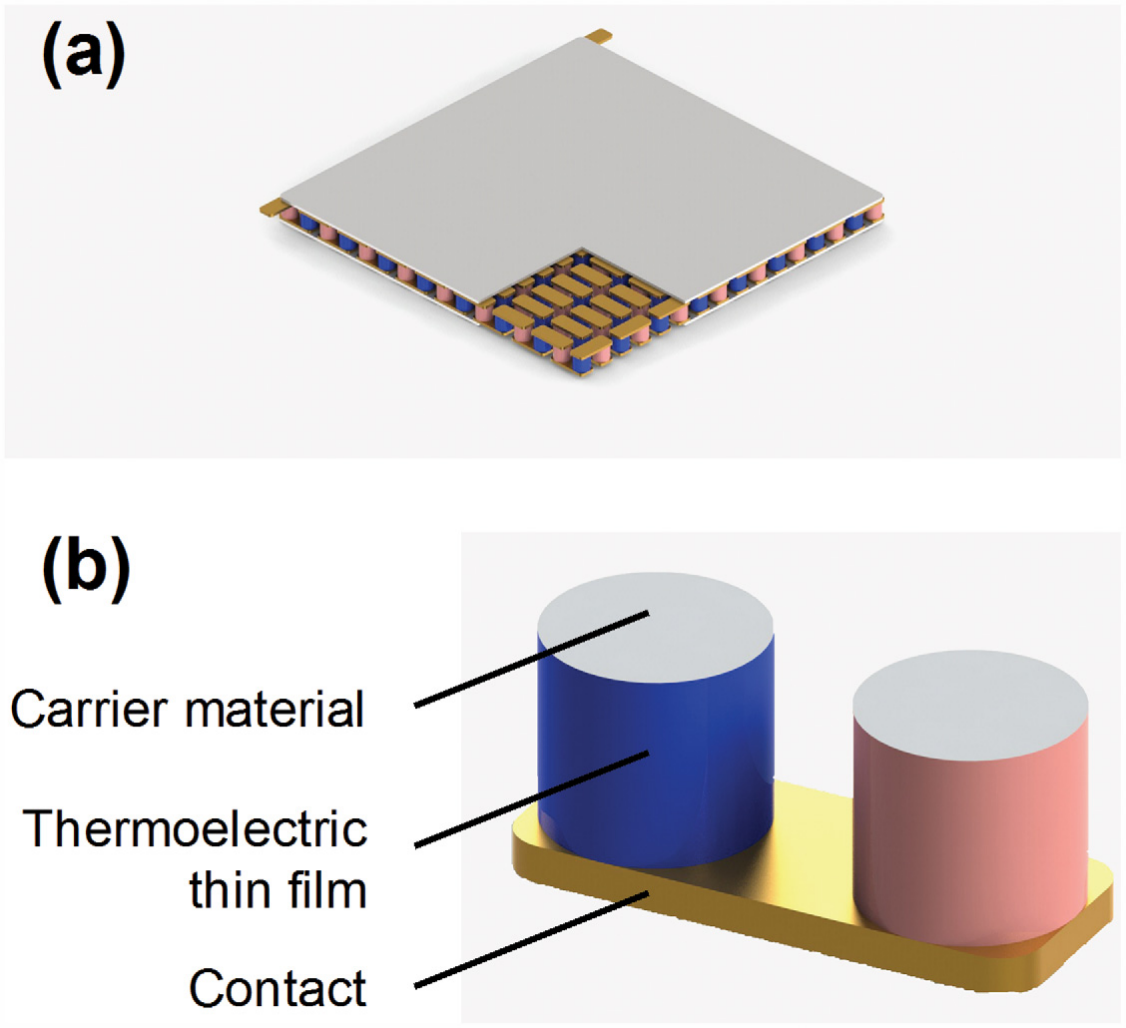
Design of a thin film thermoelectric device. (a) Rendered model of a thermoelectric device, based on the supported thin film approach. A square has been removed from the upper ceramic plate, in order to reveal the thermoelectric legs. (b) A pair of thermoelectric legs is presented in detail, where the substrate is colored grey, the p-type thermoelectric material blue, the n-type material red and the metallic contact golden. The n-type and p-type layers are situated at the surface of cylinders made from a supporting material with a low *κ*.

This deduced general knowledge can be now applied, in order to illustrate, how an actual thin film based thermoelectric device could be built. Fig 6 presents a model of a Peltier cooler, rendered using the software Solidworks. In this model, the thermoelectric legs consist from cylinders of a supporting material, which are coated with a nanometer thin layer of the corresponding p- and n-type thermoelectric materials. These cylinders could be easily produced by coating longer supporting rods with the thermoelectric material, followed by their slicing in small pieces. Due to the thickness of the cylinders, the obtained legs would possess mechanical stability; therefore they could be used in the same way as conventional thermoelectric legs. This would give the device an advantage over other types of nanostructured arrays, [82–85] such as those using silicon nanowires, [86–88] which are more difficult to produce and could suffer under mechanical stress.

Moreover, the thermoelectric legs would now correspond to the general case of thermal resistances in parallel since both the layers and the supporting cylinders are simultaneously heated. As a result, the effective thermal conductivity of the device would be even better controllable, reaching the value of the substrate for thin films, as theoretically expected (see S1 Text). Since the mass of the layers is negligible in comparison to that of the supporting material, no significant radial variation of the temperature should be observed (see Fig 3(a)–3(c)).

## Conclusions

In summary, we have investigated the different factors that can influence the heat transfer within a thermoelectric thin film. For this purpose, time dependent simulations of the temperature distribution have been made using finite element analysis, as a qualitative method to evaluate the thermal conductivity. Pyrex glass has been chosen as a substrate for the simulations, due to its broad utilization in practical investigations.

The three thermoelectric materials investigated, copper, copper(I) oxide and lead telluride, with properties ranging from metallic to semiconductive, have been used to draw general conclusions. Using this simple system, we have demonstrated that the temperature gradient within a thin film can be enhanced in two ways: either by reducing the thickness of the thermoelectric layer, or in the presence of a supporting material with a low thermal conductivity *κ*. In the latter case, the heated face should be maintained at a constant temperature for optimal performance in use. Accordingly, large temperature gradients have been observed even for highly conductive metals, such as copper, where the large *κ* usually controls the heat transfer. Furthermore, the use of a substrate with a low *κ* has reduced the effective thermal conductivity of the film-substrate ensemble, down to the value of the supporting material for nanometer thin films. We have further employed these findings to suggest a thin film based thermoelectric device, which may present improved properties in comparison to the existing alternatives.

In other words, we do not only attempt to clarify the factors influencing the thermal transfer in supported thin films to a broader audience, but we also try to offer an alternative to the common ways of making nanostructured thermoelectric devices. The next steps in the development of this approach should concentrate on its implementation in real world applications. We are looking forward to seeing further researchers implementing this strategy into their own device prototypes.

## Supporting Information

S1 Fig Thermal properties for the Pyrex glass. The thermal conductivity (*κ*) is depicted in black and the heat capacity at constant pressure (C_*P*_) in red.
(PDF)

S2 Fig Time dependence of the temperature profile for a supported PbTe layer. The view is magnified along the direction of the temperature gradient. The front face of the Pyrex substrate is heated at a constant temperature of 393 K.
(PDF)

S3 Fig Full-scale comparison of a 100 nm thin film with a 1 mm thick layer, both supported on a glass slide. The temperature is kept constant at the front face of the Pyrex glass (PG) for: (a) Cu, (b) Cu_2_O, (c) PbTe. The heat flux is maintained constant at the front face of the Pyrex glass for: (d) Cu, (e) Cu_2_O, (f) PbTe.
(PDF)

S4 Fig Full-scale comparison of a free standing 1 mm thick layer and a 1 mm layer on a microscope slide. The temperature is kept constant at the front face of the Pyrex glass (PG) for: (a) Cu, (b) Cu_2_O, (c) PbTe. The heat flux is maintained constant at the front face of the Pyrex glass for: (d) Cu, (e) Cu_2_O, (f) PbTe.
(PDF)

S5 Fig Temperature distribution within the microscope slide. The Pyrex glass (PG) is either heated at one end at a constant temperature of 393 K (marked with T in the sample name), or at a constant heat flux of 10^5^ W m^−2^ (marked with E): (a) enlarged view, (b) full-scale view.
(PDF)

S6 FigModel of our investigated system, for illustrating the different possible types of heat transfer.The thermal resistances of the layers are arranged in parallel (similar to our investigations) or in series, according to the direction of the heat flow $\dot{Q}$. y_*glass*_ represents the length of the layers, A is their heated area and *κ* the thermal conductivity. The green thermoelectric layer is indicated by index 1, while the blue supporting material has the index 2.(PDF)

S7 Fig Time dependence of the temperature distribution along the upper surface of the supported PbTe layers. The generated temperature profiles are shown once every second, for the full simulated time range of 1-40 s. The front face of the Pyrex glass is heated at a constant temperature of 393 K in the frames (a) and (b), corresponding to Fig 3(c) from the main article. The heat flux is maintained constant at the front face of the Pyrex glass for frames (c) and (d), as shown in Fig 3(f) from the main text.
(PDF)

S1 Table Fitted functions for different properties of the investigated materials, as taken from Comsol Multiphysics. (PDF)

S1 Text Mathematical deduction of the effective thermal conductivity obtained from the 1D model of thermal resistances in parallel. (PDF)
