# Supplementary material for: Decreasing the Effective Thermal Conductivity in Glass Supported Thermoelectric Layers
Source: PLoS One. 2016 Mar 16;11(3):e0151708. doi: 10.1371/journal.pone.0151708 (PMC4794206; doi:10.1371/journal.pone.0151708)
Supplement: S1 Fig — The thermal conductivity (κ) is depicted in black and the heat capacity at constant pressure (CP) in red. (PDF) [file pone.0151708.s001.pdf]

Kevin Bethke[1], Virgil Andrei[1], and Klaus Rademann[1,*]
[1]Humboldt-Universität zu Berlin, Department of Chemistry, Berlin, 12489, Germany
* klaus.rademann@chemie.hu-berlin.de

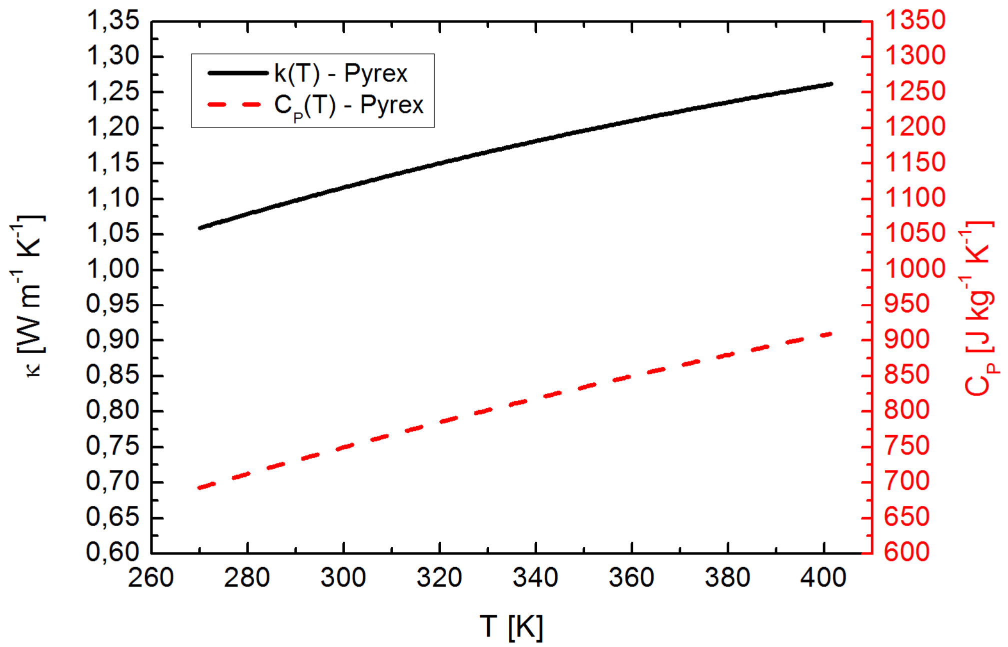

S1 Fig. Thermal properties for the Pyrex glass. The thermal conductivity (κ) is depicted in black and the heat capacity at constant pressure (C_p) in red.
