# Supplementary material for: Decreasing the Effective Thermal Conductivity in Glass Supported Thermoelectric Layers
Source: PLoS One. 2016 Mar 16;11(3):e0151708. doi: 10.1371/journal.pone.0151708 (PMC4794206; doi:10.1371/journal.pone.0151708)
Supplement: S3 Fig — The temperature is kept constant at the front face of the Pyrex glass (PG) for: (a) Cu, (b) Cu2O, (c) PbTe. The heat flux is maintained constant at the front face of the Pyrex glass for: (d) Cu, (e) Cu2O, (f) PbTe. (PDF) [file pone.0151708.s003.pdf]

Decreasing the Effective Thermal Conductivity in Glass Supported Thermoelectric Layers - Supplementary Information
Kevin Bethke[1], Virgil Andrei[1], and Klaus Rademann[1,*]
[1]Humboldt-Universität zu Berlin, Department of Chemistry, Berlin, 12489, Germany
* klaus.rademann@chemie.hu-berlin.de

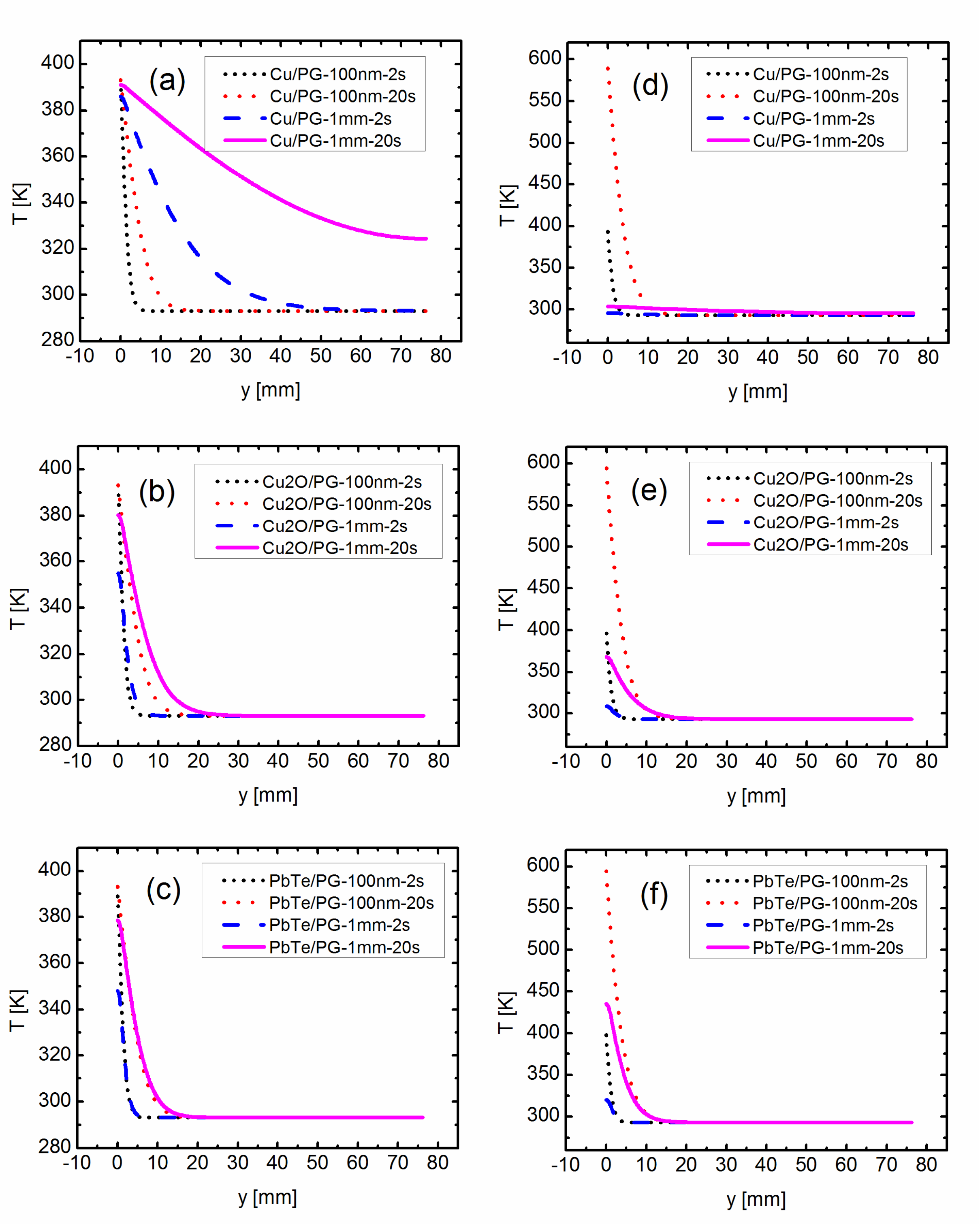

S3 Fig. Full-scale comparison of a 100 nm thin film with a 1 mm thick layer, both supported on a glass slide. The temperature is kept constant at the front face of the Pyrex glass (PG) for: (a) Cu, (b) Cu_2O, (c) PbTe. The heat flux is maintained constant at the front face of the Pyrex glass for: (d) Cu, (e) Cu_2O, (f) PbTe.
