# Supplementary material for: Decreasing the Effective Thermal Conductivity in Glass Supported Thermoelectric Layers
Source: PLoS One. 2016 Mar 16;11(3):e0151708. doi: 10.1371/journal.pone.0151708 (PMC4794206; doi:10.1371/journal.pone.0151708)
Supplement: S5 Fig — The Pyrex glass (PG) is either heated at one end at a constant temperature of 393 K (marked with T in the sample name), or at a constant heat flux of 105 W m−2 (marked with E): (a) enlarged view, (b) full-scale view. (PDF) [file pone.0151708.s005.pdf]

Decreasing the Effective Thermal Conductivity in Glass Supported Thermoelectric Layers - Supplementary Information
Kevin Bethke[1], Virgil Andrei[1], and Klaus Rademann[1,*]
[1]Humboldt-Universität zu Berlin, Department of Chemistry, Berlin, 12489, Germany
* klaus.rademann@chemie.hu-berlin.de

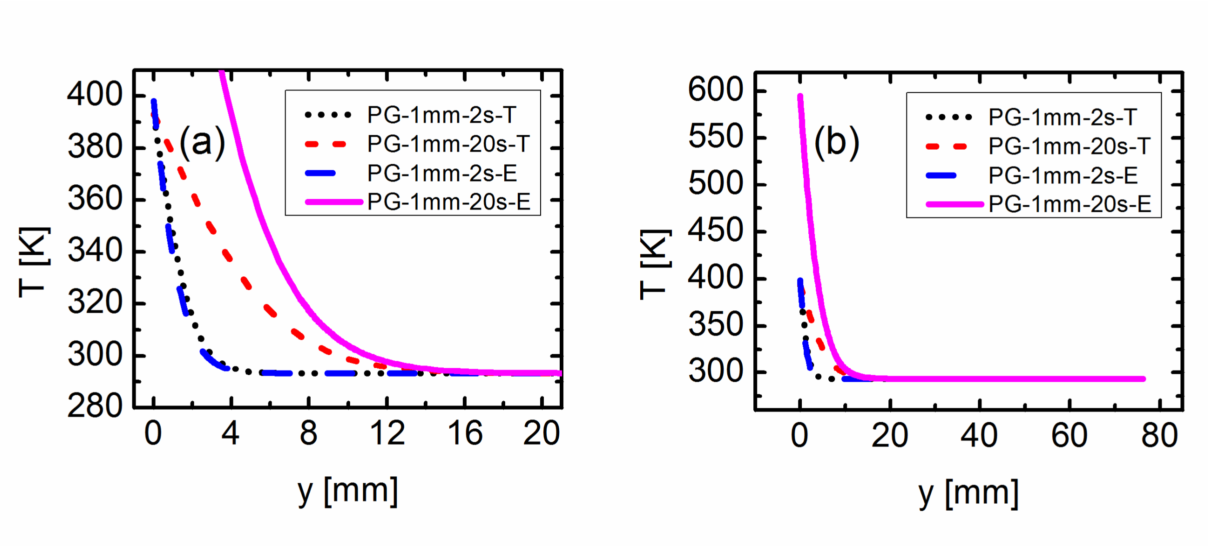

S5 Fig. Temperature distribution within the microscope slide. The Pyrex glass (PG) is either heated at one end at a constant temperature of 393 K (marked with T in the sample name), or at a constant heat flux of 10^5 W m^{-2} (marked with E): (a) enlarged view, (b) full-scale view.
