# Supplementary material for: Decreasing the Effective Thermal Conductivity in Glass Supported Thermoelectric Layers
Source: PLoS One. 2016 Mar 16;11(3):e0151708. doi: 10.1371/journal.pone.0151708 (PMC4794206; doi:10.1371/journal.pone.0151708)
Supplement: S6 Fig — The thermal resistances of the layers are arranged in parallel (similar to our investigations) or in series, according to the direction of the heat flow Q˙. yglass represents the length of the layers, A is their heated area and κ the thermal conductivity. The green thermoelectric layer is indicated by index 1, while the blue supporting material has the index 2. (PDF) [file pone.0151708.s006.pdf]

**Kevin Bethke[1], Virgil Andrei[1], and Klaus Rademann[1,*]**
[1]Humboldt-Universität zu Berlin, Department of Chemistry, Berlin, 12489, Germany
* klaus.rademann@chemie.hu-berlin.de

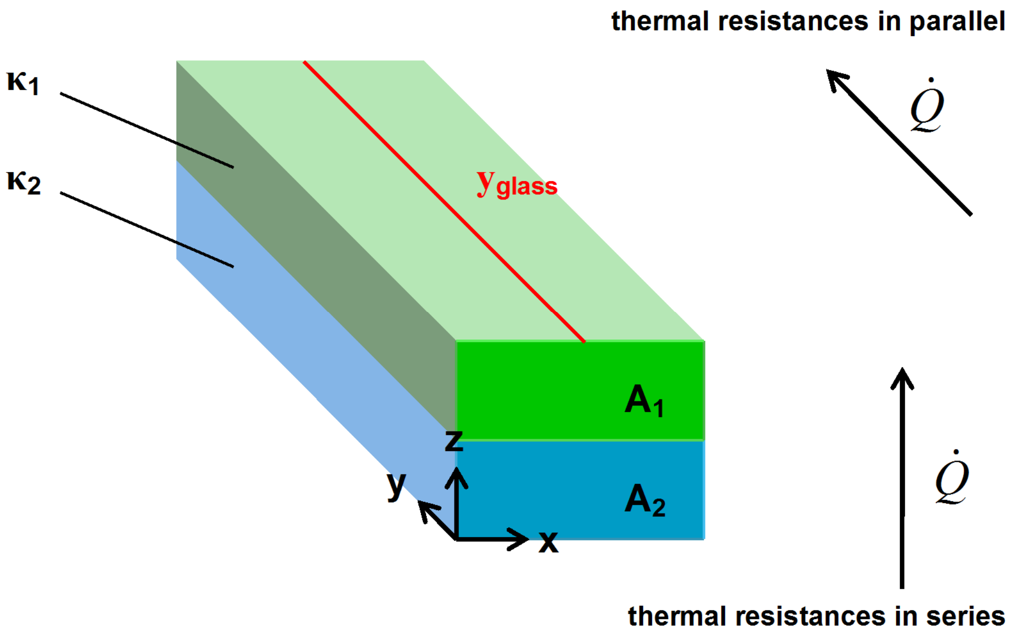


**S6 Fig. Model of our investigated system, for illustrating the different possible types of heat transfer.** The thermal resistances of the layers are arranged in parallel (similar to our investigations) or in series, according to the direction of the heat flow $\dot{Q}$. $y_{glass}$ represents the length of the layers, A is their heated area and κ the thermal conductivity. The green thermoelectric layer is indicated by index 1, while the blue supporting material has the index 2.
