# Supplementary material for: Decreasing the Effective Thermal Conductivity in Glass Supported Thermoelectric Layers
Source: PLoS One. 2016 Mar 16;11(3):e0151708. doi: 10.1371/journal.pone.0151708 (PMC4794206; doi:10.1371/journal.pone.0151708)
Supplement: S7 Fig — The generated temperature profiles are shown once every second, for the full simulated time range of 1-40 s. The front face of the Pyrex glass is heated at a constant temperature of 393 K in the frames (a) and (b), corresponding to Fig 3(c) from the main article. The heat flux is maintained constant at the front face of the Pyrex glass for frames (c) and (d), as shown in Fig 3(f) from the main text. (PDF) [file pone.0151708.s007.pdf]

Kevin Bethke[1], Virgil Andrei[1], and Klaus Rademann[1,*]
[1]Humboldt-Universität zu Berlin, Department of Chemistry, Berlin, 12489, Germany
* klaus.rademann@chemie.hu-berlin.de

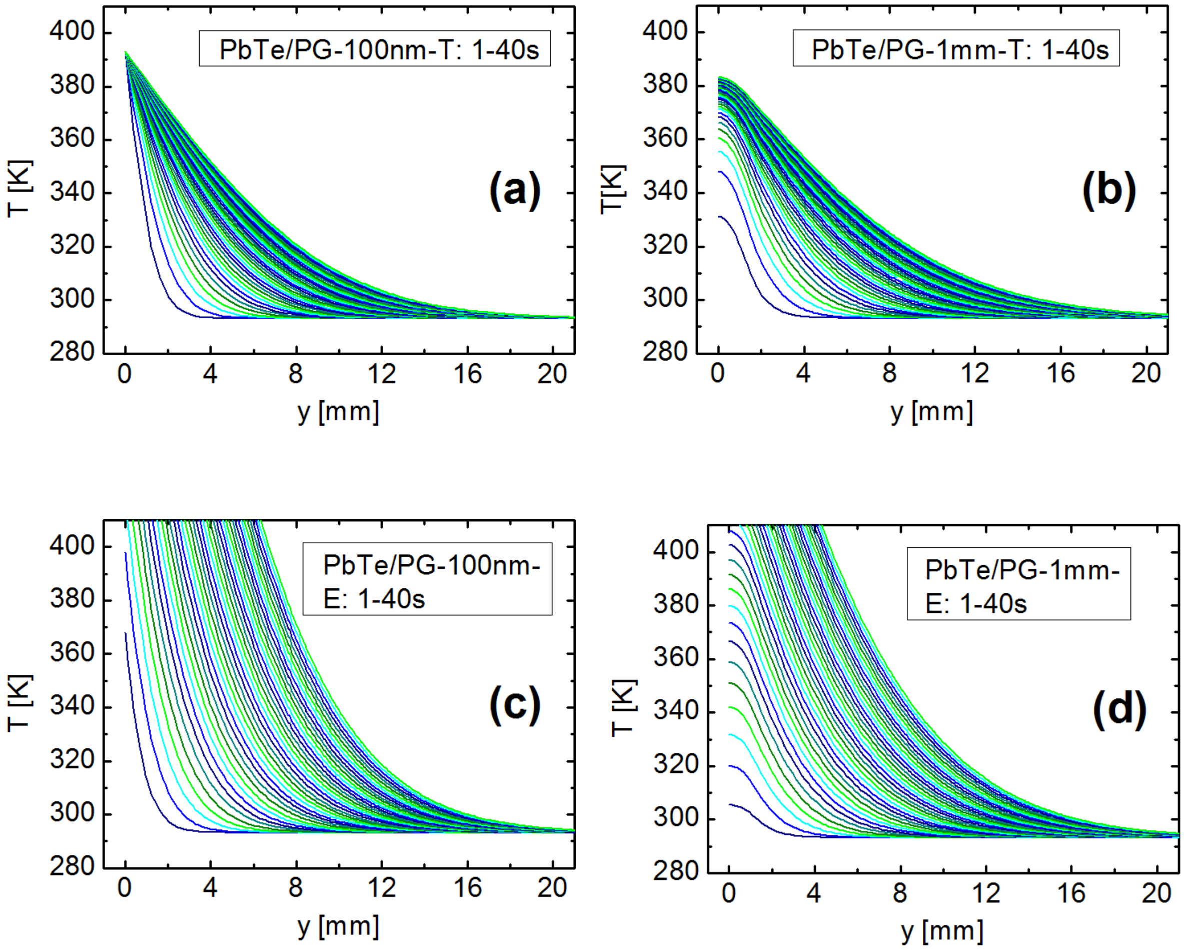

S7 Fig. Time dependence of the temperature distribution along the upper surface of the supported PbTe layers. The generated temperature profiles are shown once every second, for the full simulated time range of 1-40 s. The front face of the Pyrex glass is heated at a constant temperature of 393 K in the frames (a) and (b), corresponding to Fig. 3(c) from the main article. The heat flux is maintained constant at the front face of the Pyrex glass for frames (c) and (d), as shown in Fig. 3(f) from the main text.
