# Supplementary material for: Decreasing the Effective Thermal Conductivity in Glass Supported Thermoelectric Layers
Source: PLoS One. 2016 Mar 16;11(3):e0151708. doi: 10.1371/journal.pone.0151708 (PMC4794206; doi:10.1371/journal.pone.0151708)
Supplement: S1 Table — (PDF) [file pone.0151708.s008.pdf]

Decreasing the Effective Thermal Conductivity in Glass Supported Thermoelectric Layers - Supplementary Information
Kevin Bethke[1], Virgil Andrei[1], and Klaus Rademann[1,*]
[1]Humboldt-Universität zu Berlin, Department of Chemistry, Berlin, 12489, Germany
* klaus.rademann@chemie.hu-berlin.de

S1 Table. Fitted functions for different properties of the investigated materials, as taken from Comsol Multiphysics.

Material	Property	T_{low} [K]	T_{high} [K]	Expression
Pyrex glass	σ^{-1} [Ω m]	293	423	$\exp(144.02-0.7492484 \cdot T^1+0.001992116 \cdot T^2-2.629179E-6 \cdot T^3+1.378133E-9 \cdot T^4)$
Pyrex glass	κ [W m^{-1} K^{-1}]	90	522	$0.1448239+0.00500369 \cdot T^1-6.929144E-6 \cdot T^2+3.477568E-9 \cdot T^3$
Pyrex glass	C_P [J kg^{-1} K^{-1}]	20	700	$-54.4515+3.650568 \cdot T^1-0.003602436 \cdot T^2+1.22855E-6 \cdot T^3$
Pyrex glass	ρ [kg m^{-3}]	80	673	$2234.113-0.002096489 \cdot T^1-6.441875E-5 \cdot T^2+9.703111E-8 \cdot T^3-5.572413E-11 \cdot T^4$
Cu	σ^{-1} [Ω m]	100	1358	$-3.514582E-9+7.064722E-11 \cdot T^1-8.917638E-15 \cdot T^2+1.026538E-17 \cdot T^3$
Cu	κ [W m^{-1} K^{-1}]	100	300	$831.0-6.986667 \cdot T^1+0.04406667 \cdot T^2-1.253333E-4 \cdot T^3+1.333333E-7 \cdot T^4$
Cu	κ [W m^{-1} K^{-1}]	300	1358	$528.9926-1.034752 \cdot T^1+0.003059339 \cdot T^2-4.399776E-6 \cdot T^3+2.929135E-9 \cdot T^4$ $-7.326451E-13 \cdot T^5$
Cu	C_P [J kg^{-1} K^{-1}]	60	300	$-215.2814+8.236392 \cdot T^1-0.04732108 \cdot T^2+1.291112E-4 \cdot T^3-1.357031E-7 \cdot T^4$
Cu	C_P [J kg^{-1} K^{-1}]	300	1300	$342.764+0.1338348 \cdot T^1+5.535252E-5 \cdot T^2-1.971221E-7 \cdot T^3+1.140747E-10 \cdot T^4$
Cu	ρ [kg m^{-3}]	250	800	$9062.242-0.3913962 \cdot T^1-8.947644E-5 \cdot T^2$
Cu_2O	C_P [J kg^{-1} K^{-1}]	293	1516	$269.8289+0.759696 \cdot T^1-7.304674E-4 \cdot T^2+2.218871E-7 \cdot T^3+4.095487E-11 \cdot T^4$
Cu_2O	ρ [kg m^{-3}]	73	1573	$5993.536+0.04921465 \cdot T^1-9.434866E-5 \cdot T^2+6.27139E-9 \cdot T^3$
