# Supplementary material for: Decreasing the Effective Thermal Conductivity in Glass Supported Thermoelectric Layers
Source: PLoS One. 2016 Mar 16;11(3):e0151708. doi: 10.1371/journal.pone.0151708 (PMC4794206; doi:10.1371/journal.pone.0151708)
Supplement: S1 Text — (PDF) [file pone.0151708.s009.pdf]

Decreasing the Effective Thermal Conductivity in Glass Supported Thermoelectric Layers - Supplementary Information
Kevin Bethke[1], Virgil Andrei[1], and Klaus Rademann[1,*]
[1]Humboldt-Universität zu Berlin, Department of Chemistry, Berlin, 12489, Germany
* klaus.rademann@chemie.hu-berlin.de

S1 Text. Mathematical deduction of the effective thermal conductivity obtained from the 1D model of thermal resistances in parallel.

S6 Fig illustrates both possible types of heat transfer, for thermal resistances in parallel and in series, depending on the direction of the heat flow $\dot{Q}$. y_{glass} represents the length of the layers, A is their heated area and κ the thermal conductivity (with index 1 for the thermoelectric layers and index 2 for the supporting material). In the parallel case, both layers are heated simultaneously through the surfaces A_1 and A_2, whereas in our simulations only one of the two areas is heated at a time. Nevertheless, the solution of this similar 1D case can be used, to verify our qualitative conclusions.

The definition of the thermal resistance R is given in Eq. (S1). Analogous to an electrical resistor, the thermal resistance is inversely proportional to the thermal conductivity κ.

$$R = \frac{y_{glass}}{A \cdot \kappa} \tag{S1}$$

The same rules that apply for electrical circuits can be now used, to determine the effective thermal conductivity κ_{eff} of our simulated systems. This mathematical deduction is presented in Eq. (S2) to (S5).

$$\frac{1}{R_{eff}} = \frac{1}{R_1} + \frac{1}{R_2} \quad \Leftrightarrow \quad \frac{(A_1 + A_2) \cdot \kappa_{eff}}{y_{glass}} = \frac{A_1 \cdot \kappa_1}{y_{glass}} + \frac{A_2 \cdot \kappa_2}{y_{glass}} \tag{S2}$$

$$(A_1 + A_2) \cdot \kappa_{eff} = A_1 \cdot \kappa_1 + A_2 \cdot \kappa_2 \quad \Leftrightarrow \quad \kappa_{eff} = \frac{A_1 \cdot \kappa_1 + A_2 \cdot \kappa_2}{A_1 + A_2} \tag{S3}$$

I) If $A_1 << A_2$, $\kappa_1 > \kappa_2$ and $A_1 \cdot \kappa_1 << A_2 \cdot \kappa_2$:

$$\kappa_{eff} \cong \frac{A_2 \cdot \kappa_2}{A_2} = \kappa_2 < \kappa_1 \tag{S4}$$

In this case it is revealed that a decreasing thickness of the thermoelectric layers leads indeed to a reduction of κ_{eff}, down to the value of the substrate. This result for the parallel heat transfer is consistent with our assumptions from subsection 3.A (see also Fig. 2), where only the front surface of the supporting material is heated.

II) If $A_1 = A_2$ and $\kappa_1 > \kappa_2$:

$$\kappa_{eff} = \frac{A_2 \cdot (\kappa_1 + \kappa_2)}{2 \cdot A_2} = \frac{\kappa_1 + \kappa_2}{2} < \kappa_1 \tag{S5}$$

The simple 1D model of thermal resistances in parallel also confirms the qualitative results from subsection 3.B and Fig. 3: the presence of a substrate is beneficial for the reduction of the effective thermal conductivity, even in the case of a thick thermoelectric layer.
